# Screening and Evaluation of Polyhydroxybutyrate-Producing Strains from Indigenous Isolate *Cupriavidus taiwanensis* Strains

**DOI:** 10.3390/ijms12010252

**Published:** 2011-01-05

**Authors:** Yu-Hong Wei, Wei-Chuan Chen, Chin-Kuei Huang, Ho-Shing Wu, Yi-Ming Sun, Chi-Wei Lo, Om-Murugan Janarthanan

**Affiliations:** 1 Graduate School of Biotechnology and Bioengineering, Yuan Ze University, Chung-Li, Taoyuan 320, Taiwan; E-Mails: itispay@gmail.com (W.-C.C.); vkuei@yahoo.com.tw (C.-K.H.); murugan_jana@yahoo.co.in (O.-M.J.); 2 Department of Chemical Engineering and Materials Science, Yuan Ze University, Chung-Li, Taoyuan 320, Taiwan; E-Mails: cehswu@saturn.yzu.edu.tw (H.-S.W.); cesunym@saturn.yzu.edu.tw (Y.-M.S.); s929303@mail.yzu.edu.tw (C.-W.L.)

**Keywords:** PHB, Sudan black B staining, IR spectra, fermentation strategy, nutrient-limited conditions

## Abstract

Polyhydroxyalkanoate (PHA) is a biodegradable material with many potential biomedical applications, including medical implants and drug delivery. This study developed a system for screening production strains in order to optimize PHA production in *Cupriavidus taiwanensis* 184, 185, 186, 187, 204, 208, 209 and *Pseudomona oleovorans* ATCC 29347. In this study, Sudan black B staining, Infrared (IR) and Gas Chromatography (GC) analysis indicated that the best strain for PHA synthesis is *C. taiwanensis* 184, which obtains polyhydroxybutyrate (PHB). Cultivation of *C. taiwanensis* 184 under a pH of 7.0, at 30 °C, and at an agitation rate of 200 rpm, obtained a PHB content of 10% and PHB production of 0.14 g/L. The carbon and nitrogen types selected for analysis of PHB production by *C. taiwanensis* 184 were gluconic acid and NH_4_Cl, respectively. Optimal carbon/nitrogen ratio for PHB production was also determined. This study demonstrated a PHB content of 58.81% and a PHB production of 2.44 g/L when the carbon/nitrogen ratio of 8/1 was selected for *C. taiwanensis* 184. A two-stage fermentation strategy significantly enhanced PHB content and PHB production. Under a two-stage fermentation strategy with nutrient-limited conditions, *C. taiwanensis* 184 obtained a PHB content of 72% and a PHB concentration of 7 g/L. Finally, experimental results confirmed that optimizing the growth medium and fermentation conditions for cultivating the indigenous *C. taiwanensis* 184 strain substantially elevated PHB content from 10% to 72% and PHB production from 0.14 g/L to 7 g/L, respectively.

## 1. Introduction

Rapid population growth in recent decades has resulted in severe environmental degradation. Because conventional plastics are not degradable by microorganisms, many companies have attempted to develop biodegradable alternatives. Polyhydroxyalkanoates (PHAs) are polyesters synthesized by various microorganisms, such as *Ralstonia eutropha*, *Alcaligenes latus*, *Aeromonas hydrophila*, *Pseudomonas putida* and *Bacillus spp.* [1–5]. Because of their good biodegradability and biocompatibility, PHAs have attracted interest in their use as an alternative to petroleum-based plastics including fine chemicals, plastics, printing materials, bio-fuel [6]. Furthermore, PHA properties for biomedical applications could also be varied, based on the co-monomer structures of the copolymers [7]. The PHA types, such as polyhydroxybutyrate (PHB), poly(hydroxybutyrate-co-hydroxyvalerate) (PHBV), poly(hydroxybutyrate-co-hydroxyhexanoate) (PHBHHx) and polyhydroxyoctanoate (PHO) are frequently studied for biomedical applications including tissue regeneration devices, repair devices, repair patches and sutures [7–11]. In terms of drug delivery materials, PHAs which showed a faster drug release rate than that by ploylatic acid (PLA) are potential candidates [7]. Additionally, future studies would focus on other PHA types to reveal drug delivery properties depending on their copolymer structures [12–14]. The PHAs are energy storage materials that accumulate in bacteria under certain conditions, such as insufficient nitrogen, phosphorus, sulfur and oxygen, or excessive carbon [1–6].

The PHAs are classified by the number of carbon atoms in their monomers. In “short-chain length” PHAs, such as polyhydroxybutyrate (PHB) and polyhydroxyvalerate, carbon numbers of monomers are 3 to 5. Conversely, carbon numbers in medium chain-length PHA monomers range from 6 to 16. In addition to PHB, more than 140 different PHAs have been identified. The PHB identified in *B. megaterium* in 1926 by a French microbiologist was characterized by its large accumulations of PHB [7]. Because of its good biodegradability and biocompatibility, PHB has potential use in advanced drug delivery systems. However, PHA production strains are still needed. The lipophilic staining with Sudan Black B (SB staining) reportedly has high sensitivity in PHA screening [15]. After SB staining, bacteria containing PHAs exhibit dark granules. Therefore, Sudan Black B staining is a simple method of screening potential strains for PHAs.

The main fermentation strategies used to obtain bio-products are batch culture, fed-batch culture, continuous culture and two-stage fermentation. Two-stage fermentation is currently the most common method of producing PHAs [16]. In the first stage of the proposed process, biomass is increased to the level needed for PHB production. In the second stage, nutrients are limited in order to stimulate PHB synthesis by bacteria. This study also applied a two-stage fermentation strategy.

The aim of this study is to screen indigenous strains for PHB production. This study also evaluates the appropriate carbon and nitrogen types for PHB production and the feasibility of maximizing PHB production by optimizing growth conditions such as agitation rate, temperature and pH. Batch culture and two-stage fermentation strategies were also evaluated in terms of optimal carbon/nitrogen (C/N) ratios for PHB production.

## 2. Results and Discussions

### 2.1. The Selection of PHB Production Strain

**Figure 1:**
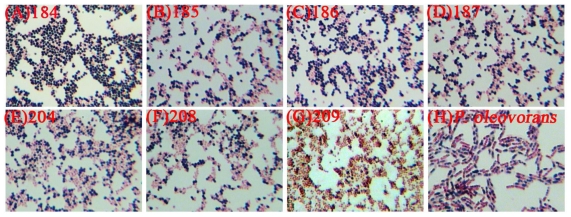
Sudan black B stain of PHB granules (black section) on *C. taiwanensis* and *P. oleovorans* ATCC 29347 observed under 100X oil immersion objective.

**Figure 2:**
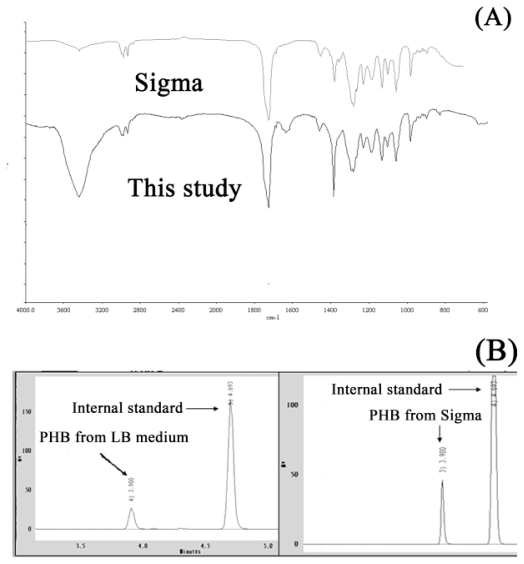
Comparison of IR spectra of PHB standard obtained from Sigma with PHB from MM9 medium (**A**) and GC analysis of PHB standard obtained from Sigma and PHB from LB medium (**B**).

The local indigenous strains, *i.e.*, *C. taiwanensis* 184, 185, 186, 187, 204, 208, 209 and *Pseudomonas oleovorans* ATCC 29347 were evaluated to select the PHA production strain. The PHA production and PHA type were verified by SB staining, Gas Chromatography (GC) and Infrared (IR), respectively. The PHA granules could exactly indicate the PHA production ability of microorganisms [15]. According to the results of SB staining, the *C. taiwanensis* 184 showed significant PHA granules (black section) over other stains (Figure 1). Furthermore, the PHA production of the local indigenous strains was further evaluated by GC. Among the eight strains, *C. taiwanensis* 184 also showed the highest PHA production of 0.14 g/L and PHA content of 10% more than the other strains (Table 1). According to the above mentioned results, this study suggested that *C. taiwanensis* 184 should be the production strain used. The purified PHA extracted from the culture broth of *C. taiwanensis* 184 was analyzed by IR and GC. The IR spectrum of the purified PHA product shows two mainly intense absorption peaks at 1720 cm ^−1^ and 1280 cm ^−1^ corresponding to C=O and C-O functional groups, respectively (Figure 2A). Hence, the purified PHA extracted from the culture broth of *C. taiwanensis* 184 should be polyester. Moreover, the GC spectrum of the purified PHA product also showed two major peaks occurred at retention time 3.9 minutes and 4.6 minutes, which corresponds to the internal standard (benzoic acid) and PHB standard purchased from Sigma, respectively (Figure 2B). These results (see Figure 2) also indicated that the strain, *C. taiwanensis* 184, was a potential strain for producing PHB and hence this strain was selected.

### 2.2. Effect of Growth Condition on PHB Production by *C. taiwanensis* 184

#### 2.2.1. Effect of Initial Culture pH

**Figure 3:**
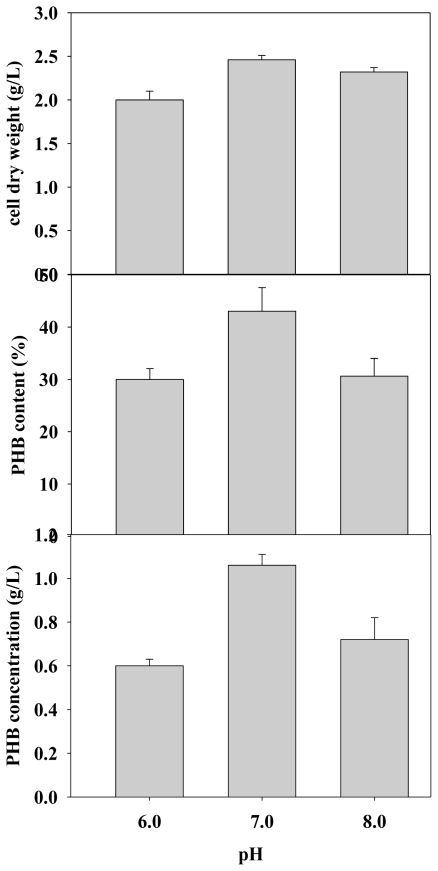
Effects of various pH values on PHB production with *C. taiwanensis* 184 (n = 3).

Typically, metabolic processes are highly susceptible to even slight changes in pH. Therefore, proper control of pH is critical. Figure 3 shows the effect of initial culture pH on cell dry weight (CDW) and PHB production. The experimental results showed that the initial pH, which was controlled with Defined M9 medium (DM9), could be adjusted by adding concentrated hydrochloric acid or sodium hydroxide. Figure 3 clearly shows that an initial pH of 7.0 obtained the highest PHB content (43.04%). These results were consistent with Palleroni and Palleroni [17], who recommended a pH range of 6.0 to 7.5 for microbial growth and PHB production. Although PHB production can be controlled by precisely manipulating pH, the experimental data indicated that pH values other than 7 affect PHB production. These results suggested that PHB production is sensitive to the pH of cultivation.

#### 2.2.2. Effect of Culture Temperature

**Figure 4:**
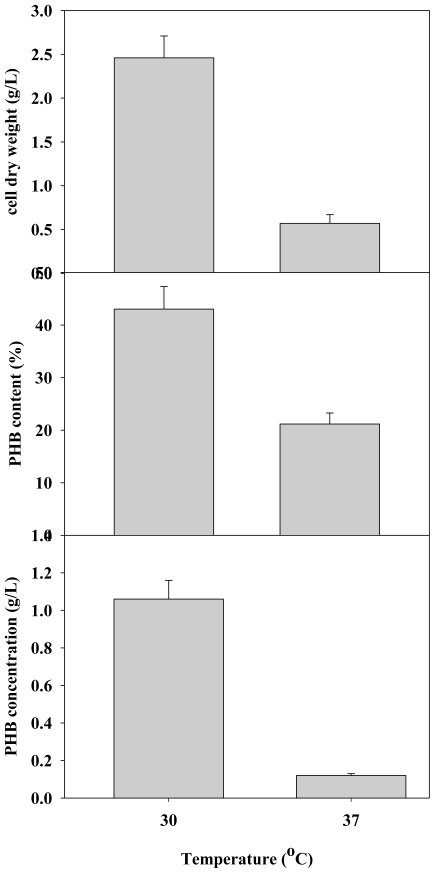
Effects of various temperatures on PHB production with *C. taiwanensis* 184 (n = 3).

Because temperature, pH and agitation rate all affect dissolved oxygen levels and mass transfer efficiency, these environmental factors profoundly affect cellular growth and bioproduct production. Thus, the effects of temperature on CDW and PHB production by *C. taiwanensis* 184 were also evaluated. In this work, PHB production by *C. taiwanensis* 184 was examined at 30 °C and 37 °C. Figure 4 shows that the optimal temperature for cell growth and PHB production was 30 °C. The highest CDW of 1.06 g/L and PHB production of 1.06 g/L occurred at 30 °C.

#### 2.2.3. Effect of Agitation Rate

**Figure 5:**
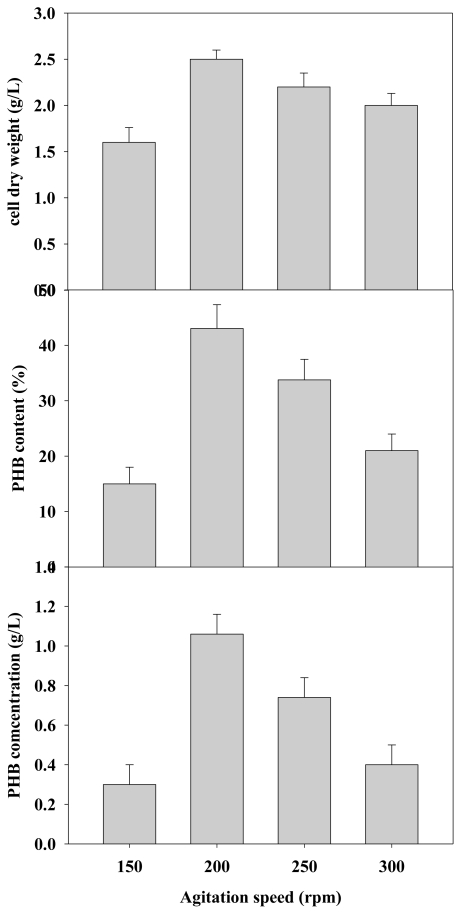
Effects of various agitation rates on PHB production with *C. taiwanensis* 184 (n = 3).

The effect of agitation rate on cell growth and PHB production was also analyzed. The experimental results showed that the CDW and PHB production in the batch culture increased, respectively, and agitation rate increased from 150 to 200 rpm. Therefore, an elevated agitation rate apparently enhanced both cell growth and PHB production from 0.2 g/L to 1.05 g/L (Figure 5). Limiting agitation rate to 200 rpm also slightly decreased PHB production from 1.05 g/L to 0.4 g/L, probably because of the excessive shear force produced at agitation speeds exceeding 250 rpm.

### 2.3. Media Optimization

#### 2.3.1. Effect of Carbon and Nitrogen Types on PHB Production by *C. taiwanensis* 184

Exactly how different carbon and nitrogen types affect PHB production was evaluated using various carbon sources (including carbohydrates, e.g., glucose, food-level glucose, lactose, raffinose, sucrose, galactose, dulcitol, and mannitol; and hydrocarbons, e.g., ethanol, gluconic acid, oxalic acid, malic acid, and galacturonic acid); and various nitrogen sources, e.g., NH_4_Cl, CH_3_COONH_4_, NH_4_NO_3_, (NH_4_)_2_SO_4_, and NH_2_CONH_2_. The carbohydrates were sterilized separately by filtration and added to defined M9 medium at a final concentration of 1% (w/v). Various carbon and nitrogen sources at concentrations of 4 g/L and 1 g/L, respectively, were used as the substrate to evaluate their effects on PHB accumulation in defined M9 media. The cultivation was performed at a temperature of 30 °C and an agitation rate of 200 rpm under aerobic conditions. The *C. taiwanensis* 184 strain was examined for PHB production in different carbon and nitrogen sources. Among these tests in this study, gluconic acid supported PHB synthesis. However, other than glucose, carbon sources did not enhance PHB synthesis. Conversely, although all nitrogen sources positively affected PHB synthesis, NH_4_Cl had the largest effect. Hence, Table 2 shows that the medium with gluconic acid and NH_4_Cl as the carbon and nitrogen source, respectively, apparently had a positive effect on PHB production. Thus, the final composition of the modified M9 medium (MM9 medium) was as follows: Gluconic acid: 4 g/L, NH_4_Cl: 1 g/L, Na_2_HPO_4_: 7 g/L, NaH_2_PO_4_: 3 g/L, 0.01 M CaCl_2_: 10 mL and 0.1 M MgSO_4_·7H_2_O: 10 mL.

#### 2.3.2. Effect of Carbon/Nitrogen Ratio on PHB Production by *C. taiwanensis* 184

In living organisms, carbon (C) requirements are generally larger than nitrogen (N) requirements. The balance of these elements (C/N) determines how bacteria use an organic material [18]. To enhance PHB production, the C/N ratios of 1/1, 2/1, 4/1, 8/1 and 20/1 in MM9 medium were compared to determine the optimal ratio. Table 3 shows that a C/N ratio of 8/1 obtained the highest values for CDW (4.15 g/L), PHB production (2.44 g/L), and PHB content (58.81%). These results suggest that a C/N ratio of 8/1 is optimal for the accumulation of PHB by *C. taiwanensis* 184. However, at ratios above 8/1, PHB production consistently increased up to a ratio of 20/1, at which point the C/N ratio decreased. The PHB production by *C. taiwanensis* 184 was 0.89 g/L when PHB content was 52.77% (Table 3), and C/N ratio was 20/1. In addition, PHB production by *C. taiwanensis* 184 also decreased at C/N ratios higher or lower than 8/1. These results are attributable to high concentration of carbon source that involves the substrate, and *C. taiwanensis* 184 is inhibited, reducing CDW, PHB content and PHB production. Additionally, high C/N and low C/N ratios might affect the physiological conditions of the microorganisms, including cell proliferation and polymerization of PHB [18]. Therefore, the optimal C/N ratio of 8/1 was used in subsequent experiments.

### 2.4. Two-Stage Fermentation in 5 L Fermenter

**Figure 6:**
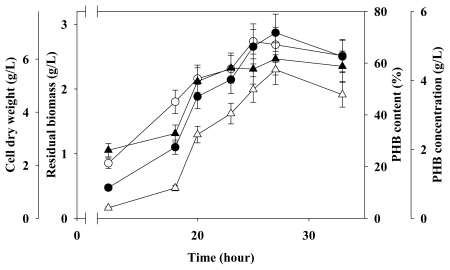
Time course of cell growth and PHB production with *C. taiwanensis* 184 in a 5-liter fermenter (culture conditions: initial pH = 7, temperature = 30 °C, agitation rate = 200 rpm, carbon/nitrogen ratio = 8:1; (n = 3)). Closed circle: CDW; Open circle: Residual biomass; Closed triangle: PHB content; Open triangle: PHB concentration.

To enhance PHB accumulation, two-stage fermentation strategies were used. To date, few reports have specifically analyzed nutrient limitation conditions. In recent years, two-stage fermentation has become the preferred strategy for enhancing PHA production [16,19–21]. Therefore, this study also applied the two-stage fermentation strategy to evaluate PHB production in *C. taiwanensis* 184. Figure 6 shows the time course of PHB production under the above optimized conditions in a 5-liter fermenter with a 3-liter volume. Initially, PHB production was 3.8 g/L, PHB content was 62%, CDW was 7.2 g/L, and residual biomass was 3.2 g/L. By the end of the fermentation process, the results of PHB production, PHB content, CDW and residual biomass were 3.3 g/L, 59%, 6 g/L and 2.5 g/L, respectively. These experimental results indicate that the optimal duration of the second stage experiment was approximately 28 hours.

**Figure 7:**
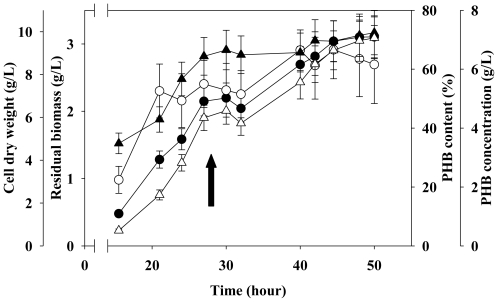
Time course of PHB production, residual biomass and CDW by *C. taiwanensis* 184 using nutrient-limiting strategy (n = 3). Closed circle: CDW; Open circle: Residual biomass; Closed triangle: PHB content; Open triangle: PHB concentration. The arrow indicates the starting time for the second stage.

The two-stage fermentation strategy was performed under nutrient-limited conditions to stimulate PHB production by *C. taiwanensis* 184. This study indicated that increasing biomass is essential for PHB production in the first stage. The nutrient limitation conditions in the second stage then cause the bacteria to synthesize PHB. The optimal nutrient limitation strategy for enhancing PHB production, reportedly limits magnesium, phosphorous and sulfur [22–26]. This study also evaluated the effects of NaH_2_PO_4_ and MgSO_4_·7H_2_O. The best limitation condition for PHB production was half magnesium-limitation (0.05 M) rather than phosphate-limitation or sulfate-limitation at the second stage (data not shown). In all nutrient limitation conditions, CDW slightly increased when magnesium concentration was halved. Furthermore, the residual biomass maintained at a constant level (shown in Figure 7) and the PHB production increased, which was attributed to intracellular accumulation of PHB at the second stage. In the second stage, biomass on MM9 media gradually increased for up to 50 hours after cultivation under all limitation conditions. Interestingly, when magnesium was limited but not completely depleted, PHB content in the MM9 media was 72%, and PHB production was 7 g/L. The above results indicate that, when magnesium is limited, crude biomass increases from 7 g/L to 10 g/L in the first stage of fermentation, and PHB production increases during the second stage of fermentation. These results also suggest that magnesium may be crucial to the production of PHB by *C. taiwanensis* 184. The limitation on cellular growth in the second stage may prompt the *C. taiwanensis* 184 to store PHB as an energy source based on a physiological adaptation [24]. The medium that was used in the second stage might prompt *C. taiwanensis* 184 to store polymer during this period of physiological adaptation [24]. This observation is consistent with reports that limiting nitrogen sharply increases the PHB content of *A. latus* from 52% to 83% [23].

## 3. Experimental Section

### 3.1. Microorganisms Screening for PHB Production

Eight indigenous bacterial strains screened from Taiwan for PHB production were *C. taiwanensis* 184, *C.* taiwanensis 185, *C. taiwanensis* 186, *C. taiwanensis* 187, *C. taiwanensis* 204, *C. taiwanensis* 208 and *C. taiwanensis* 209 were identified by phylogenic analysis of the 16S rDNA and *P. oleovorans* ATCC 29347 purchased from Bioresource Collection and Research Center, Food Industrial Research and Development Institute, Taiwan. A loopful of each culture taken from a slant was inoculated into 2 mL growth medium containing beef extract (Sigma, St. Louis, MO): 1.0 g/L, peptone (Sigma): 5.0 g/L, yeast extract (Sigma): 2.0 g/L, NaCl (Sigma): 5.0 g/L, with a pH of 7.0. For the experiments, 2 mL of inoculum was inoculated into 98 mL liquid medium and cultivated at 30 °C and 200 rpm after 12-h cultivation and incubated for 24 hours in an aerobic environment. The cultures were aseptically centrifuged at 2000 × *g* for 30 min to separate the biomass. The biomass was then inoculated into flasks containing the production media described below and grown for 48 hours at 30 °C and at 200 rpm on a rotary shaker. To identify the best PHB production stain, Sudan black B staining was performed, and PHA synthesis was measured by GC and IR spectral (IFS28; Bruker, Bemen, Germany) analysis.

### 3.2. PHB Production Media and Conditions

In the basally-defined M9 medium (DM9 medium) used to experimentally cultivate the strains, the media content was as follows: 4 g/L goucose (Sigma), 1 g/L NH_4_Cl (Sigma), 7 g/L Na_2_HPO_4_ (Sigma), 3 g/L NaH_2_PO_4_ (Sigma), 10 mL 0.01 M CaCl_2_ (Sigma) and 10 mL 0.1 M MgSO_4_·7H_2_O (Sigma). Various carbon and nitrogen sources were also selected for evaluating PHB production. Exactly how carbon and nitrogen types affected PHB production was evaluated by analyzing various carbon sources (including carbohydrates, e.g., Glucose, Food-level glucose, Lactose, Raffinose, Sucrose, Galactose, Dulcitol, Mannitol), hydrocarbons, e.g., Ethanol, Gluconic acid, Oxalic acid, Malic acid and Galacturonic acid), and various nitrogen sources (e.g., NH_4_Cl (Sigma), CH_3_COONH_4_ (Sigma), NH_4_NO_3_ (Sigma), (NH_4_)_2_SO_4_ (Sigma) and NH_2_CONH_2_ (Sigma)). To investigate the effects of temperatures (30 °C and 37 °C), pHs (6–8) and agitation rates (150–300 rpm) on PHB production, the batch culture was performed, respectively. In these experiments, batch fermentation was performed in a 500 mL shake flask containing 100 mL of culture medium. Culture medium components and fermentation conditions varied according to the experimental design.

### 3.3. Quantifications of Cell Growth and PHB

Cell growth was monitored by turbidity at a 600 nm optimal density. Cell concentration defined as dry cell weight per liter of culture broth and PHB concentration, was determined as previously described [12]. Residual gluconic acid test was estimated by HPLC column (Aminex HPX-87H, BIO-RAD), Mobile phase: 5mM H_2_SO_4_ (Sigma), flow rate: 0.5 mL/min, RI detector: L-2490 (Hitachi, Tokyo, Japan).

### 3.4. Two-Stage Fermentation Strategy for PHB Production

The PHB synthesis was analyzed under varying fermentation conditions, including pH, temperature, and agitator speed. The PHB production in the 5 L fermenter was evaluated to determine the proper conditions for cultivating *C. taiwanensis* 184. Additionally, PHB production in the 5 L fermenter was evaluated when using a two-stage fermentation strategy. After increasing cell concentration in the first stage, a nutrient limitation strategy was used for PHB production in the second stage as described elsewhere [16,19–21].

### 3.5. Staining of Bacteria with Sudan Black B

Several drops of microbial broth were fixed on a glass slide by applying heat and then stained with a 3% Sudan Black B (w*/*v in 70% ethanol, Sigma) solution for 10 minutes. The slide was then immersed in xylene until completely decolonized. The sample was counterstained with safranin (5% w*/*v in distilled water, Sigma) for 10 seconds, washed again with distilled water, and dried. After adding several drops of immersion oil directly to the completely dry slide, the cells were examined by optical microscopy (Leica, Tokyo, Japan) (15).

### 3.6. Analytical Methods

The GC analysis was performed after methanolyzing the polymer in sulfuric acid and methanol asas described by Lutke *et al.* [27]. Internal standard benzoic acid was used to measure PHB concentration by comparing PHB peaks. The infrared (IR) spectra of polymer samples were analyzed according to Lutke *et al.* [27]. The purity of the standard PHB that was purchased from Sigma is defined as 100%. Based on this definition, the relative purity of the purified PHB, as used in the GC and IR analyses, was determined to be around 95%.

## 4. Conclusion

Screening of the indigenous strain *Cupriavidus taiwanensis* 184 by Sudan Black B staining confirmed its potent PHB production. Moreover, PHB production in MM9 medium was 4.15 g/L when measured under the following conditions: Temperature of 30 °C, pH of 7 and agitation speed of 200 rpm. To enhance PHB accumulation, a two-stage fermentation strategy was used. This study showed that such a strategy significantly enhanced PHB content and PHB production. Under a two-stage fermentation strategy with nutrient-limited conditions, *C. taiwanensis* 184 obtained a PHB content of 72% and a PHB concentration of 7 g/L. However, further research is needed to determine the commercial potential of *C. taiwanensis* 184 for producing long-chain biodegradable polymers. The findings of this study provide a reference for further research in the use of PHB for manufacturing biodegradable polymers.

## Figures and Tables

**Table 1:** Comparison of PHB content and PHB concentration among the eight strains (n = 3).

Strains	PHB content (%)	PHB concentration (g/L)
*C. taiwanensis* 184	9.67 ± 0.11	0.15 ± 0.03
*C. taiwanensis* 185	3.43 ± 0.09	0.07 ± 0.01
*C. taiwanensis* 186	2.08 ± 0.02	0.09 ± 0.02
*C. taiwanensis* 187	0.45 ± 0.06	0.03 ± 0.01
*C. taiwanensis* 204	0.54 ± 0.08	0.04 ± 0.01
*C. taiwanensis* 208	0.25 ± 0.03	0.01 ± 0.01
*C. taiwanensis* 209	0.63 ± 0.06	0.02 ± 0.01
*P. oleovorans* ATCC 29347	0.09 ± 0.01	0.03 ± 0.01

**Table 2:** Effects of various carbon and nitrogen sources on PHB synthesis (n = 3).

Carbon types				Nitrogen types	

Carbohydrates		Hydrocarbons		Inorganic nitrogen	

Carbon source	PHB production	Carbon source	PHB production (g/L)	Nitrogen source	PHB production (g/L)
Glucose	+	Ethanol	−	NH_4_Cl	++
Glucose (food)	+	Gluconic acid	++	CH_3_COONH_2_	+
Lactose	−	Oxalic acid	−	NH_4_NO_3_	+
Raffinose	−	Malic acid	−	(NH_4_)_2_SO_4_	+
Sucrose	−	Galacturoni c acid	−	NH_2_CONH_2_	+
Galactose	−				
Dulcitol	−				
Mannitol	−				

**Table 3:** Effects of carbon/nitrogen ratio on the synthesis of PHB (n = 3).

C/N (mol/mol)	CDW (g/L)	PHB Concentration (g/L)	PHB Content (%)
1/1	1.42 ± 0.28	0.42 ± 0.05	29.21 ± 2.33
2/1	2.42 ± 0.62	1.04 ± 0.12	43.10 ± 3.15
4/1	2.45 ± 0.57	1.20 ± 0.14	48.86 ± 2.19
8/1	4.15 ± 0.91	2.44 ± 0.36	58.81 ± 3.93
20/1	1.69 ± 0.43	0.89 ± 0.02	52.77 ± 2.62

## References

[1] Chakraborty P Gibbons W Muthukumarappanl K Conversion of volatile fatty acids into polyhydroxyalkanoate by *Ralstonia eutropha* J. Appl. Microbiol 2009 106 1996 2005 19320958 10.1111/j.1365-2672.2009.04158.x

[2] Yu PH Chua H Huang AL Ho KP Conversion of industrial food wastes by *Alcaligenes latus* into polyhydroxyalkanoates Appl Biochem Biotechnol 1999 77–79 445 454 10.1385/abab:78:1-3:445 15304714

[3] Zhang HF Ma L Wang ZH Chen GQ Biosynthesis and characterization of 3-hydroxyalkanoate terpolyesters with adjustable properties by *Aeromonas hydrophila* Biotechnol. Bioeng 2009 104 582 589 19517520 10.1002/bit.22409

[4] Ren Q de Roo G Witholt B Zinn M Thöny-Meyer L Influence of growth stage on activities of polyhydroxyalkanoate (PHA) polymerase and PHA depolymerase in *Pseudomonas putida* U BMC Microbiol 2010 10 254 20937103 10.1186/1471-2180-10-254 PMC2959000

[5] Singh M Patel SK Kalia VC *Bacillus subtilis* as potential producer for polyhydroxyalkanoates Microb. Cell Fact 2009 8 38 19619289 10.1186/1475-2859-8-38 PMC2719590

[6] Chanprateep S Current trends in biodegradable polyhydroxyalkanoates J. Biosci. Bioeng 2010 110 621 632 20719562 10.1016/j.jbiosc.2010.07.014

[7] Chen GQ A microbial polyhydroxyalkanoates (PHA) based bio- and materials industry Chem. Soc. Rev 2009 38 2434 2446 19623359 10.1039/b812677c

[8] Dong Y Li P Chen CB Wang ZH Ma P Chen GQ The improvement of fibroblast growth on hydrophobic biopolyesters by coating with polyhydroxyalkanoate granule binding protein PhaP fused with cell adhesion motif RGD Biomaterials 2010 31 8921 8930 20728212 10.1016/j.biomaterials.2010.08.001

[9] Cousley RR A stent-guided mini-implant system J. Clin. Orthod 2009 43 403 407 19684361

[10] Xu XY Li XT Peng SW Xiao JF Liu C Fang G Chen KC Chen GQ The behaviour of neural stem cells on polyhydroxyalkanoate nanofiber scaffolds Biomaterials 2010 31 3967 3975 20153524 10.1016/j.biomaterials.2010.01.132

[11] Han I Shim KJ Kim JY Im SU Sung YK Kim M Kang IK Kim JC Effect of poly(3-hydroxybutyrate-co-3-hydroxyvalerate) nanofiber matrices cocultured with hair follicular epithelial and dermal cells for biological wound dressing Artif Organs 2007 31 801 808 18001389 10.1111/j.1525-1594.2007.00466.x

[12] Yilgor P Hasirci N Hasirci V Sequential BMP-2/BMP-7 delivery from polyester nanocapsules J. Biomed. Mater. Res A 2010 93 528 536 19585564 10.1002/jbm.a.32520

[13] Zhu XH Wang CH Tong YW In vitro characterization of hepatocyte growth factor release from PHBV/PLGA microsphere scaffold J. Biomed. Mater. Res A 2009 89 411 423 18431776 10.1002/jbm.a.31978

[14] Xiong YC Yao YC Zhan XY Chen GQ Application of polyhydroxyalkanoates nanoparticles as intracellular sustained drug-release vectors J. Biomater. Sci. Polym. Ed 2010 21 127 140 20040158 10.1163/156856209X410283

[15] Burdon KL Fatty materials in bacteria and fungi revealed by staining dried, fixed slide preparations J. Bacteriol 1946 52 665 678 10.1128/jb.52.6.665-678.1946 PMC518253 16561232

[16] Hartmann R Hany R Witholt B Zinn M Simultaneous biosynthesis of two copolymers in *Pseudomonas putida* GPo1 using a two-stage continuous culture system Biomacromolecules 2010 11 1488 1493 20459087 10.1021/bm100118t

[17] Palleroni NJ Palleroni AV Alcaligenes latus, a new species of hydrogen-utilizing bacteria Int. J. Syst. Bacteriol 1978 28 416 424

[18] Chanprateep S Katakura Y Visetkoop S Shimizu H Kulpreecha S Shioya S Characterization of new isolated *Ralstonia eutropha* strain A-04 and kinetic study of biodegradable copolyester poly(3-hydroxybutyrate-co-4-hydroxybutyrate) production J. Ind. Microbiol. Biotechnol 2008 35 1205 1215 18712546 10.1007/s10295-008-0427-5

[19] Sun Z Ramsay JA Guay M Ramsay B Increasing the yield of MCL-PHA from nonanoic acid by co-feeding glucose during the PHA accumulation stage in two-stage fed-batch fermentations of *Pseudomonas putida* KT2440 J. Biotechnol 2007 132 280 282 17442441 10.1016/j.jbiotec.2007.02.023

[20] Albuquerque MG Eiroa M Torres C Nunes BR Reis MA Strategies for the development of a side stream process for polyhydroxyalkanoate (PHA) production from sugar cane molasses J. Biotechnol 2007 130 411 421 17602776 10.1016/j.jbiotec.2007.05.011

[21] Liu HY Hall PV Darby JL Coats ER Green PG Thompson DE Loge FJ Production of polyhydroxyalkanoate during treatment of tomato cannery wastewater Water Environ. Res 2008 80 367 372 18536488 10.2175/106143007x221535

[22] Lee SY Wong HH Choi J Lee SH Lee SC Han CS Production of medium-chainlength polyhydroxyalkanoates by high-cell-density cultivation of Pseudomonas putida under phosphorus limitation Biotechnol. Bioeng 2000 68 466 470 10745215

[23] Valappil SP Rai R Bucke C Roy I Polyhydroxyalkanoate biosynthesis in *Bacillus cereus* SPV under varied limiting conditions and an insight into the biosynthetic genes involved J. Appl. Microbiol 2008 104 1624 1635 18194257 10.1111/j.1365-2672.2007.03678.x

[24] Wang F Lee SY Poly(3-hydroxybutyrate) production with high productivity and high polymer content by a Fed-batch culture of Alcaligenes latus under nitrogen limitation Appl Environ Microbiol 1997 63 3703 3706 16535699 10.1128/aem.63.9.3703-3706.1997 PMC1389255

[25] Albuquerque MG Torres CA Reis MA Polyhydroxyalkanoate (PHA) production by a mixed microbial culture using sugar molasses: effect of the influent substrate concentration on culture selection Water Res 2010 44 3419 3433 10.1016/j.watres.2010.03.021 20427069

[26] Shang L Jiang M Chang HN Poly(3-hydroxybutyrate) synthesis in fed-batch culture of *Ralstonia eutropha* with phosphate limitation under different glucose concentrations Biotechnol. Lett 2003 25 1415 1419 14514042 10.1023/a:1025047410699

[27] Lütke-Eversloh T Bergander K Luftmann H Steinbüchel A Identification of a new class of biopolymer: bacterial synthesis of a sulfur-containing polymer with thioester linkages Microbiology 2001 147 11 19 11160796 10.1099/00221287-147-1-11

